# Vaccine Development for Human Pneumoviruses

**DOI:** 10.3390/vaccines13060569

**Published:** 2025-05-26

**Authors:** Elhadji Birane Mboup, Marie-Ève Hamelin, Julia Dubois, Manuel Rosa-Calatrava, Guy Boivin

**Affiliations:** 1 Infectious and Immune Diseases Research Program, CHU de Québec-Université Laval Research Center, Québec, QC G1V 4G2, Canada; elhadji-birane.mboup@crchudequebec.ulaval.ca (E.B.M.); marie-eve.hamelin@crchudequebec.ulaval.ca (M.-È.H.);; 2 International Research Laboratory RESPIVIR France-Canada, Centre de Recherche en Infectiologie, Faculté de Médecine RTH Laennec, 69008 Lyon, France, Université Claude Bernard Lyon 1, Lyon, France, CHU de Québec-Université Laval Research Center, Québec, QC G1V 4G2, Canada; julia.dubois@univ-lyon1.fr; 3 CIRI, Centre International de Recherche en Infectiologie, Team VirPath, Univ Lyon, Inserm, U1111, Université Claude Bernard Lyon 1, CNRS, UMR5308, ENS de Lyon, 69007 Lyon, France; 4 Virnext, Faculté de Médecine RTH Laennec, Université Claude Bernard Lyon 1, Université de Lyon, 69008 Lyon, France; 5 Department of Pediatrics, Faculty of Medicine, Université Laval, Québec, QC G1V 0A6, Canada

**Keywords:** pneumovirus, respiratory syncytial virus (RSV), human metapneumovirus (HMPV), lower respiratory tract infection (LRTI), vaccine, clinical trial

## Abstract

Background: Pneumoviruses are etiologic agents of respiratory tract infections and a major cause of morbidity and mortality worldwide, particularly affecting young children, the elderly, and individuals with underlying clinical conditions. These viruses are associated with a significant burden, particularly in low- and middle-income countries, where reported deaths attributable to respiratory syncytial virus (RSV) and human metapneumovirus (HMPV) in young children are important. Recent developments have been noted in the prevention of pneumoviral infections. Method: In this review, we analyzed clinical trials of the approved RSV vaccines, as well as the recent prominent platform technologies used in RSV vaccine research. In addition, we discussed combination vaccines targeting RSV, HMPV, and Human Parainfluenza Virus Type 3 (HPIV3) that have entered clinical trials. Results: Recent advancements include the approval of three RSV vaccine candidates: AREXVY^®^(GSK), ABRYSVO^®^(Pfizer), and mRESVIA^®^(Moderna). These vaccines are primarily intended for older adults, with ABRYSVO^®^ also capable of providing passive immunization to infants via maternal administration. The review highlights RSV vaccine platform technologies and combination vaccines currently being evaluated in clinical settings. Conclusions: While significant progress has been made in RSV vaccine development, especially with three approved candidates, the development of vaccines for HMPV remains an unmet medical need. Ongoing research in combination vaccines holds promise for broader protection against multiple respiratory viruses in the future.

## 1. Introduction

Acute respiratory tract infections caused by viral pathogens have been recognized as a global public health threat and remain one of the leading causes of death [1]. According to the Global Burden of Disease (GBD) 2021 data, lower respiratory tract infections (LRTIs) have a death rate of 28.7 per 100,000, making them the seventh leading infectious cause of death worldwide in 2021. This ranking excluded COVID-19, which has radically impacted global mortality rates and was recognized as the second leading global cause of death [2]. LRTIs—affecting the bronchi, bronchioles, and alveoli—are a significant cause of mortality among infants and the elderly. Bronchiolitis in infants and pneumonia across all age groups are the primary contributors to LRTI-related mortality [3]. Human pneumoviruses, consisting of respiratory Syncytial Virus (RSV) and human metapneumovirus (HMPV), are key contributors to LRTI across a range of age groups and underlying diseases, with RSV being the most common cause of infections and hospitalizations in children under 2 years of age [4], with 3.3 million cases of LRTI and 101,400 deaths in children aged 0–60 months in 2019 [5]. Severe disease in older adults can worsen prior conditions and increase hospital admission and mortality rates [6]. Even though HMPV infections are typically less severe than those caused by RSV [7], their incidence is comparable to that of influenza and parainfluenza viruses. For instance, HMPV contributes to a significant number of hospitalizations each year, with an estimated 502,000 admissions among children under 5 in 2018 [8]. Outbreaks of RSV/HMPV in long-term care facilities are responsible for high mortality and morbidity rates in the elderly [9,10]. Given the public health burden caused by RSV and HMPV, these two viruses are considered prime targets for the development of safe and effective vaccines. While there are currently no prophylactic modalities available for HMPV, monoclonal antibodies and market-approved vaccines targeting the viral fusion protein have been developed to prevent RSV infections. This review aims to cover recent developments in RSV vaccines, with an emphasis on the market-approved ones, as well as combined vaccines, including HMPV and/or Human Parainfluenza Virus Type 3 (HPIV3) in the clinical pipeline.

## 2. Pneumoviruses

Pneumoviruses are a family of enveloped, non-segmented, negative-sense, single-stranded, and linear RNA viruses in the order *Mononegavirales*. Formerly a subfamily within the *Paramyxoviridae* family, these viruses were reclassified in 2016 in the *Pneumoviridae* family with two genera, *Orthopneumovirus* (including RSV) and *Metapneumovirus* (including HMPV) [11]. RSV was first reported from chimpanzees in 1956, where it was initially referred to as the Chimpanzee Coryza Agent [12]. It was later identified in infants suffering from severe lower respiratory tract diseases (LRTD) [13]. The first identification of HMPV was reported in the Netherlands in 2001 [14], but the virus has been circulating unrecognized for decades due to its clinical manifestation being similar to that of other respiratory viruses and difficulties with its propagation in cell cultures [15].

### 2.1. Genome

**Figure 1:**
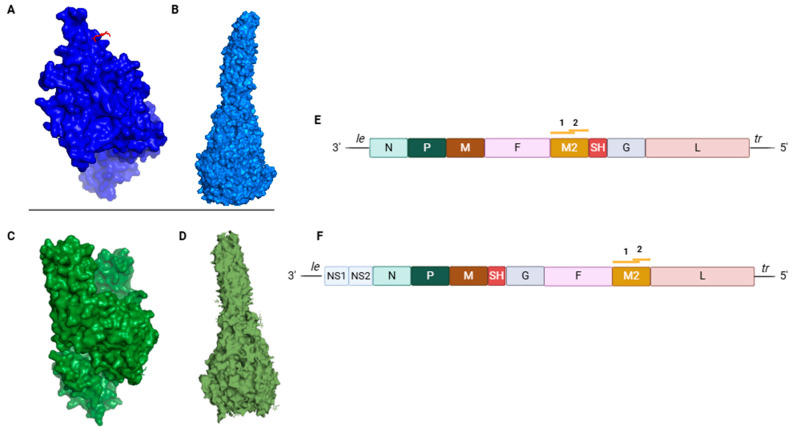
Schematic representation of prefusion (**A**) RSV-F (PDB ID: 8W3P) or (**B**) postfusion RSV-F (PDB ID: 3RRT); schematic representation of (**C**) prefusion HMPV-F PDB ID: 7SEJ) or (**D**) postfusion HMPV-F (PDB ID: 7m0i); genome organization of (**E**) HMPV or (**F**) RSV. Created with PyMOL and BioRender.

RSV genomic RNA is approximately 15.2 kb in length, with 10 genes in the order 3′-NS1-NS2-N-P-M-SH-G-F-M2-L-5′, encoding for 11 proteins. In contrast, HMPV has a slightly smaller genome of around 13.2 kb, consisting of eight genes in the order 3′-N-P-M-F-M2-SH-G-L-5′, which encode for nine proteins [16]. While both orthopneumoviruses and metapneumoviruses encode similar sets of proteins, their genome organization differs, particularly in the arrangement of the envelope genes (attachment-G, fusion-F, and small hydrophobic-SH) (Figure 1). Orthopneumoviruses contain two additional genes, NS1 and NS2, located upstream of the N gene, which encode proteins that suppress the synthesis and activity of host type 1 and type 3 interferon responses and also inhibit apoptosis [17]. Both viruses possess an M2 gene, which produces two proteins, M2-1 and M2-2, via overlapping open reading frames [18]. The genome is transcribed processively from the 3′ end by the virion-associated polymerase complex. Gene expression exhibits a polar gradient, with decreasing levels of transcription as the distance from the 3′ end increases. Transcription is guided by short (9–13 nt) conserved transcription start and termination/polyadenylation signals flanking each gene [17,19].

### 2.2. Epidemiology

RSV and HMPV outbreaks exhibit seasonal patterns worldwide [5,8]. In the northern hemisphere, RSV outbreaks generally occur from October or November to April or May of the following year, with peak activity in January or February. In the southern hemisphere, RSV infections typically peak during the winter months, from May to September, with the highest incidence often seen between May and July. The seasonal distribution of HMPV often mirrors that of RSV, although it can peak in late winter or early spring, depending on the region [20].

Each year, the two primary subtypes of RSV, RSV/A and RSV/B, along with various genotypes of each, either dominate or co-circulate during the RSV epidemic season [21,22], although infections with subtype A are potentially more frequently reported than those caused by subtype B [23,24,25]. A tendency for higher disease severity has been attributed to RSV/A; however, in more recent years, the severity between RSV/A and RSV/B subtypes has been similar [22].

A study by Cantú-Flores et al. examined global and continental patterns of RSV circulation from the first report of human infection up to the end of 2020 and identified two primary RSV circulation patterns: continuous seasons dominated by RSV/A and alternating seasons in which both subtypes predominate [26]. These patterns were consistent across various regions, although the dominant RSV strain shifted globally from year to year. The most prevalent RSV/A genotype was NA1 (including ON1 strains), which accounted for 76.3% of cases, while the dominant RSV/B genotype was BA, making up 70.65%. Notably, a global predominance of these genotypes was observed around 2014 [22]. Although multiple RSV genotypes circulated simultaneously over time, the genetic diversity among these strains has declined since 2000 [26]. Resulting from nucleotide duplication in the G gene [27,28], ON1 and BA9 have notably emerged as the predominant lineages for RSV/A and RSV/B, respectively; however, the debate about whether these lineages should be classified as distinct new genotypes is still ongoing [29,30,31].

HMPV is classified into two antigenically distinct groups: A and B [32]. Based on variations in the G and F genes, each group is further subdivided into two subgroups: A1, A2, B1, and B2 [33]. Phylogenetic analysis of the A2 subgroup based on the F gene has identified additional clades, including A2a, A2b, and A2c [34,35]. Notably, multiple subgroups of HMPV have often been found to co-circulate in the regions under investigation, whereas the precise relationship between disease severity and HMPV genotype remains unclear [36,37]. No consistent evidence supports the predominance of one subgroup over another, with several studies reporting the cocirculation of different subtypes or shifts in genotype prevalence across seasons; however, HMPV/A2 has been largely reported [37,38,39,40,41].

Implementation of non-pharmaceutical interventions (NPIs) due to COVID-19 led to changes regarding the epidemiology of respiratory viruses, such as a decrease in the circulation of RSV and HMPV during the pandemic [42,43,44]. Resurgence and off-season occurrence of RSV cases were observed after the relaxation of NPIs with more severe symptoms in naïve infants [20,45,46].

Both RSV and HMPV are etiologic agents of respiratory tract infections in people of all ages, but young children under 5 years of age, the elderly, and people with weakened immune systems are more likely to experience severe cases [47,48]. The primary risk factor for severe disease in childhood is young age, with infants in their first weeks of life being at the highest risk [49]. However, HMPV infections are less common and tend to occur in slightly older children compared to RSV [50,51,52]. Additional factors that increase the likelihood of severe illness include sex, household crowding, premature birth, and pre-existing lung conditions or congenital heart disease [53,54]. Coinfections with both viruses are reported due to the overlapping of their seasonality, and reinfections are recurrent because of waning immunity throughout time [20,41,55]. The risk of severe disease in adults is increased by the presence of underlying chronic pulmonary disease, circulatory conditions, functional disabilities, and higher viral loads. Both viruses are also a nosocomial threat to hospitalized young infants, elderly, immunocompromised, and vulnerable individuals, with fatal infections in some cases [7,15,56,57].

### 2.3. Clinical Manifestations

RSV and HMPV are spread from person to person via respiratory droplets. The incubation period can vary from 3 to 5 days for HMPV [58] or 2 to 8 days for RSV, with a mean incubation of 4 to 6 days [48]. Typical symptoms include cough, rhinorrhea, sore throat, and fever, as well as lower respiratory tract manifestations such as wheezing, difficulty in breathing, and hypoxia [47,50]. Fever has been reported to be more frequent in young patients with HMPV infections [51]. Both viruses cause upper and lower respiratory tract infections, including bronchiolitis and pneumonia [7,47]. The onset of infection in infants and young children may cause severe bronchiolitis that can sometimes be fatal. Repeated upper respiratory tract infections are common in other age groups and range from subclinical infections to symptomatic upper respiratory tract diseases. In older adults aged 65 years and over, infections lead to an increase in hospitalization and mortality [56]. Asthma exacerbations and acute exacerbations of chronic obstructive pulmonary diseases (COPD) are also observed [41,59].

### 2.4. Disease Burden

LRTIs are a leading cause of death and disability, particularly in children younger than 5 years old [5]. Around 15–50% of initial RSV infections in infants and young children affect the lower respiratory tract [60]. On a global scale estimate, RSV infections affected 24.8 million individuals in 2016, leading to approximately 76,000 deaths [61]. In 2019, it caused about 3.3 million cases of LRTI and 101,400 deaths in children under 60 months of age, while in infants aged 0–6 months, there were 6.6 million RSV-LRTI and 45,700 deaths [5]. Low- and middle-income countries are more heavily affected, accounting for over 95% of RSV-LRTI and over 97% of deaths across all age groups [5]. In the European Union, an average of 158,229 (95%CI, 140,865–175,592) RSV-associated hospitalizations are estimated among adults on an annual basis, with 92% of these hospitalizations occurring in adults ≥ 65 years [62]. In the US, the annual attack rate for older adults generally ranges between 3 and 10%, resulting in an estimate of over 177,000 hospitalizations and 14,000 deaths [63]. In children < 5 years, an average annual RSV-associated hospitalization rate of 4.0 per 1000 has been reported, with an even higher value among children 0–2 months old (23.8 per 1000) [64].

The annual cost of RSV-LRTI among the 3.7 million US infants < 12 months, totaling 592,700, is estimated at USD 1.6 billion [65]. A study by Zhang et al. in 2020 reported that the estimated global cost of managing RSV-LRTI in young children was around €4.82 billion, making RSV-related diseases a significant economic burden on healthcare systems, governments, and society [66]. Nonetheless, according to Global Burden Disease 2021 data, there was a notable decrease of 63.2% in global RSV cases, which went down to 4.59 million, along with a 66.7% decrease in RSV deaths [5]. These declines are attributable to the implementation of non-pharmaceutical interventions during the COVID-19 pandemic, which limited the transmission of respiratory viruses [67,68]. Moreover, the recent development of efficacious monoclonal antibodies and vaccines targeting the fusion protein to avert RSV infections could play a significant role in reducing RSV burden in the future, with substantial health and economic benefits [69,70,71,72].

HMPV is predominantly observed in the pediatric population, particularly among older children, and seroprevalence studies indicate that nearly all are infected by the age of 5 [73]. This virus accounts for 6.1–6.4% of acute lower respiratory infections in patients younger than 20 years old [74,75]. Although there is less global data assessing the burden of HMPV infections compared to RSV, several studies across the world have reported substantial HMPV rates in hospitalized adults and young children: India (5%) [76], China (4.08%) [77], Kenya (4.1%) [78], France (3%) [79], Japan (22.6%) [80], New Zealand (4.3%) [81]. A first 2018 global burden estimate of HMPV-related acute LRTI in young children revealed that 11.1 million cases were attributed to HMPV, resulting in 502,000 hospital admissions and 11,300 deaths worldwide. Notably, about 58% of the hospitalizations involved infants under 12 months of age, while 64% of the deaths that occurred in hospitals were among infants under 6 months. Furthermore, 79% of these deaths were reported in low- and middle-income countries [8].

## 3. Fusion Protein

Viral glycoproteins anchored within viral envelopes contain specialized regions (peptides or loops), which are crucial for merging cell and viral membranes [82].

The fusion glycoproteins of pneumoviruses are part of the class I viral fusion protein family, processed through a cleavage step to generate an amino-terminal fusion peptide that mediates viral entry during infection [83]. The F protein is predominantly displayed on the viral surface alongside G glycoprotein and small hydrophobic (SH) protein, representing a primary target for immune response and, hence, for vaccine development [84,85].

The F glycoproteins of RSV and HMPV exhibit structural similarities, sharing approximately 30% amino acid sequence (Figure 1). To enable fusion, they transition from a biologically inactive precursor (F_0_) to a mature, cleaved, and metastable homotrimer (F_1_-F_2_). They possess the property to initiate membrane fusion without an additional attachment protein [86].

In contrast to many class I fusion proteins, the 574 amino acids RSV-F_0_ undergoes cleavage at two polybasic sites (RARR109 and KKRKRR136) by furin-like proteases, yielding an internal peptide of 27 amino acids and two subunits F_1_ and F_2_ [87,88]. Even though P27 is believed to be absent in the metastable prefusion F, it has been reported on the surface of infected cells, suggesting the possibility of a partial cleavage [89]. The functional metastable prefusion protein is obtained by the trimerization of an F_1_-F_2_ disulfide bond-linked heterodimer [90]. Unlike RSV, HMPV F_0_ lacking p27 is cleaved at a unique cleavage site by (serine) proteases such as TMPRSS2, generating F_1_ and F_2_ subunits which further trimerize into a functional prefusion HMPV-F [91,92,93]. The membrane-anchored, lollypop-shaped trimer consists of a membrane-proximal coiled-coil prefusion stalk and a single globular head domain [94].

During the membrane fusion process, the fusion peptide located at the N terminus of F_2_ inserts into the cell membrane, bridging the two membranes. It occurs when a refolding leads to fusion, followed by the transition of F into a highly stable postfusion form [90,92,95]. In this final conformation, F trimers assume an overall cone shape [96].

The major antigenic sites of RSV-F described to date are Ø, I, II, III, IV, and V. Site Ø, located at the apex, is the target of the therapeutic monoclonal antibody (mAb) nirsevimab (Beyfortus^®^, Sanofi, Paris, France) [97] and is specific to the prefusion conformation, along with the proximal site V [84]. Additionally, the most highly potent neutralizing antibodies (nAbs) elicited in response to natural infection (>60%) are directed against these sites exposed in prefusion conformation, making them ideal targets for vaccine development [98]. Human mAbs binding site I are reported weakly neutralizing and are almost post-fusion specific [99]. Sites II, III, and IV are present in both conformations. Prophylactic mAb palivizumab (Synagis^®^, AstraZeneca, Cambridge, UK) and the investigational clesrovimab (MK-1654, Merck, Darmstadt, Germany) bind to site II and site IV, respectively, and prevent fusion [100,101]. Site III is highly conserved between RSV and HMPV and is targeted by the cross-neutralizing mAb MPE8 and the potent mAb RSV-199 [102,103,104]. Based on established nAbs, similar antigenic sites—Ø, I, II, III, IV, V—have been identified for HMPV [105]. Sites I, II, III, and IV have prefusion and postfusion dual tropism [106,107]. Similar to RSV-F, sites Ø and V are prefusion-specific and represent potent neutralizing sites [108]. However, in contrast to RSV-F, the most frequent potent nAbs identified target epitopes in site IV, followed by sites III and II. In addition, there are HMPV nAbs targeting sites located in regions that span adjacent antigen sites (I and III, I and IV, II and III, II and V, and III and V), highlighting immunogenicity discrepancy of the F antigen between the two viruses [109]. Additionally, over 80% of nAbs against HMPV target F epitopes on both conformations[109]. However, the prefusion structure elicits higher neutralizing titers compared to the postfusion for certain genotypes [110].

## 4. RSV Vaccines

The prevention of RSV infection has been, until recently, an unmet medical need since its first recognition as a human pathogen. Immunization strategies focus on infants, children, pregnant women, and older adults because these groups are more vulnerable to this viral infection [111]. The aim of vaccination against RSV is primarily to prevent severe RSV-associated lower respiratory tract infection (RSV-LRTI) [112]. The initial failure to develop an effective vaccine based on inactivated virus has had a lasting impact on RSV vaccine development research [113]. However, significant progress has been made, and numerous innovative vaccine platforms have been developed since then for active immunization against RSV (Table 1). These include subunit vaccines, Live Attenuated Vaccines (LAV), vector-based vaccines, and mRNA vaccines [114].

### 4.1. Inactivated Vaccines

The journey of RSV vaccine development for use in humans began in the 1960s with a formalin-inactivated and alum-adjuvanted RSV vaccine (FI-RSV) grown in monkey kidney tissue culture. Upon vaccination, however, infants developed far more severe RSV infections than non-vaccinees, and this exaggerated response to natural infection has been termed enhanced respiratory disease (ERD) [113,120]. Serological analyses from patients who suffered from ERD showed that despite the development of anti-RSV binding antibodies, their neutralizing and fusion-inhibiting responses were weak [121,122], possibly making these infants more vulnerable to severe RSV infections. Additionally, post-mortem lung tissue samples from the two lethal cases revealed immune complex deposition and complement activation, suggesting that the weak neutralizing antibodies could have heightened the risk of severe disease [123]. Additionally, marked Th2 cell activity (IL-4, IL-5, and IL-13)-related symptoms, pulmonary eosinophilia, and neutrophilic alveolitis were noted in various animal models [124,125,126,127]. This pattern was similar to the lung histopathology observed in the infants who died following RSV infection, implying that an excessive reaction to the vaccine may also have played a role in the severe complications [123]. This early disappointment, along with concerns about the potential occurrence of FI-RSV-related ERD, hindered vaccine development.

Decades later, a purified β-propiolactone inactivated RSV vaccine (BPL-RSV) adjuvanted with CpG ODN (TLR-9 ligand) and L18-MDP (NOD2 receptor ligand) was developed and evaluated in Balb/c mice. The results indicated that BPL-RSV activated APCs effectively in vitro and elicited local IgA, along with enhanced Th1-type IgG2a responses in vivo following mucosal immunization. TLR9/NOD2 ligands’ addition facilitated the affinity maturation of RSV-specific IgG antibodies and shifted T-cell responses toward increased IFN-γ production, thus suggesting a Th1-dominated response. This immunization approach provided better protection against viral challenges without ERD occurrence, as evidenced by the lack of eosinophil infiltration in the lungs. It also suggests a potential administration approach for inactivated vaccines without priming ERD [128], which needs to be assessed more closely.

### 4.2. Subunit Vaccines

In the aftermath of the failure of the FI-RSV vaccine to provide protection against infection, attention shifted to understanding the immune response to RSV. This ultimately led to the discovery of the fusion protein [129]. Antibodies directed against this protein were found to confer protection from RSV infection through passive immunization in animal models [130]. In the 1990s, the potential for passive protection against severe RSV disease by administration of immunoglobulins directed against F antigen led to the successful development of the humanized monoclonal antibodies palivizumab (Synagis^®^, AstraZeneca) [101,131] for RSV prevention in high-risk infants [132] then nirsevimab in July 2023 (Beyfortus ^®^, Sanofi) [97,133]. The ability of genetically engineered chimeric FG glycoprotein subunit vaccine to circumvent ERD was demonstrated in the primate model [125], suggesting their potential use as a safer alternative to FI-RSV. Although these vaccines were revealed to be immunogenic and well tolerated in humans, they lack protective efficacy against severe disease in vulnerable groups [134,135,136,137,138]. More recently, research on active immunization has experienced a resurgence of interest with the discovery of the stabilization of the metastable conformation of the F fusion protein by McLellan et al. [139]. Their structure-based design of a stabilized RSV-F protein exposing site Ø was a game changer and facilitated the improvement of a robust RSV immune response. This breakthrough prompted the development of several subunit RSV vaccines targeting the prefusion state, ultimately resulting in two vaccines currently approved to prevent RSV-LRTI (Table 1) [140,141,142,143].

#### 4.2.1. AREXVY^®^-RSVPreF3 OA (GSK3844766A)

On 3 May 2023, the US Food and Drug Administration (FDA) approved the world’s first RSV vaccine for individuals aged 60 years and older and subsequently for people aged 50–59 years who are at increased risk of RSV-LRTD [140,141,144]. AREXVY^®^ is an intramuscularly delivered, single-dose subunit vaccine developed by GSK, created through stabilization of the RSV/A2-F protein in its trimeric prefusion form (RSVPreF3) combined with Adjuvant System 01 (AS01_E_) as an immunostimulant [145]. A phase 1/2 study involving young and older adults demonstrated strong potency and an excellent safety profile for the AS01_E_ adjuvant combined with the highest dose of RSVPreF3 (120 μg), which emerged as the preferred formulation (RSVPreF3 OA) for further studies [146]. In a phase 3 clinical trial, an unadjuvanted formulation of the vaccine was administered to pregnant women to prevent infections in their newborns up to six months. However, the GRACE trial was put on hold in February 2022 following the emergence of safety concerns regarding higher preterm births in vaccinees than in the placebo group [147]. The late evaluation phases of the RSVPreF3 OA vaccine in older adults demonstrated an acceptable safety profile (except one report of Guillain–Barré syndrome in an ongoing study [148]) with high efficacy against RSV-LRTD (82.6%), severe RSV-LRTD (94.1%), and RSV-associated acute respiratory infection (RSV-ARI) (71.7%) over one RSV season, regardless of RSV subtypes and pre-existing conditions [115]. Furthermore, this vaccine provided effective protection in older adults over two seasons with one dose, presenting an efficacy of 67.2% against RSV-LRTD and 78.8% against severe RSV-LRTD. Nonetheless, a second dose of vaccine did not seem to improve efficacy [116]. The coadministration of RSVPreF3 OA with quadrivalent seasonal influenza vaccines (FLU-QIV, FLU-QIV-HD, and FLU-aQIV) demonstrated an acceptable safety profile in older adults, with no clinically significant interference observed in the immune responses to either vaccine. Additionally, the immune response was not inferior when comparing sequential administration [149,150,151]. Studies assessing its coadministration with a COVID-19 mRNA vaccine (Omicron XBB.1.5), a pneumococcal vaccine (PCV20), and a herpes zoster subunit vaccine (HZ/su) are ongoing. An observational study among Medicare beneficiaries ≥ 65 years, conducted between May 2023 and July 2024, suggests an increased risk of Guillain–Barré syndrome 42 days following vaccination with AREXVY^®^. However, the observation is considered insufficient to establish a causal relationship [144].

#### 4.2.2. ABRYSVO^®^-RSVPreF

ABRYSVO^®^, approved in May 2023, is a bivalent prefusion F vaccine (RSVpreF) developed by Pfizer. This intramuscularly administered single-dose vaccine contains 60 μg of trimeric F glycoproteins from both major RSV subgroups (A and B) engineered for stability in the prefusion conformation. It is indicated for active immunization of pregnant women at 32–36 weeks of gestational age for the prevention of RSV-LRTD and severe LRTD in infants from birth through 6 months of age, and also for active immunization of individuals 60 years of age and older and individuals from 18 to 59 years who are at increased risk of RSV-LRTD [152]. Phase 1/2 studies showed good safety, tolerability, and immunogenicity with no benefit of adding either Al(OH)3 [153] or CpG/Al(OH)3 [154] as adjuvants in adults and healthy older adults, respectively. Phase 2 evaluations revealed an efficacy of 86.7% in preventing symptomatic RSV infections in adults in a challenge study [155] and efficient transplacental antibody transfer with immunization in the late second or third trimester of pregnancy [156]. The phase 3 MATISSE clinical trial assessed the efficacy and safety of RSVpreF in preventing RSV-associated lower respiratory tract illness in infants through maternal immunization between 24 and 36 months of gestation. Vaccine efficacy against medically attended severe LRTI within 90 days and 180 days after birth were 81.8% and 69.4%, respectively, with no safety concerns reported. Success criteria regarding protection against medically attended RSV-associated LRTI was not met with an efficacy of 57.1% within 90 days after birth [118]. The RENOIR study evaluated vaccine efficacy in older adults. It prevented RSV-associated lower respiratory tract illness with efficacies of 66.7% and 85.7%, defined by presenting at least two signs or symptoms and three signs or symptoms, respectively. Protection regarding RSV-associated acute respiratory illness was 62.1% without any evident safety issues [117]. The administration of ABRYSVO^®^ with tetanus, diphtheria, and pertussis vaccine (Tdap) and seasonal inactivated influenza vaccine (SIIV) were evaluated. Coadministration of RSVpreF with both vaccines was found to be safe, and non-inferiority was demonstrated. However, SIIV response was lower, and an inferior immune response was only seen for acellular pertussis antigen in the case of Tdap [157,158]. The MONET study assessing the vaccine in adults at increased risk of severe RSV disease has been recently completed [159]. Furthermore, the MORISSOT study, evaluating vaccine effectiveness in pregnant participants with HIV and their infants, along with a study assessing its coadministration with nirsevimab has been launched [160,161]. Regarding AREXVY^®^, the observational study suggests an increased risk of Guillain–Barré syndrome during the 42 days following vaccination with ABRYSVO^®^, but the correlation is considered insufficient [152].

### 4.3. Live Attenuated Vaccines

Live Attenuated RSV vaccines are expected to provide numerous advantages for immunizing infants and young children. These vaccines do not prime enhanced RSV disease and effectively stimulate both systemic and local immune responses, including innate, humoral, and cellular immunity. Additionally, they can be administered intranasally and have the ability to replicate in the upper respiratory tract of young infants, even in the presence of maternal antibodies [162]. Since the initial efforts to generate LAVs for RSV through repeated passages or chemical mutagenesis [163,164,165,166], significant progress has been made using reverse genetics [167]. This technology has enabled the rational design of LAVs by deleting or modifying specific genes, resulting in a balanced profile of attenuation and immunogenicity. Notably, preclinical studies including Non-Human Primate reported that the ΔM2-2 deletion inactivates viral replication and increases transcription, while the ΔNS2 deletion optimizes the induction of innate immunity, along with G and SH deletions [168,169,170]. Several candidates have been developed, and pooled data from seven phase 1 clinical trials indicate an estimated efficacy of 67% and 88% against medically attended acute RSV illness and medically attended LRTI, respectively [171].

A promising candidate, RSV/ΔNS2/Δ1313/I1314L, sponsored by NIAID, is undergoing phase 3 clinical trial. The RSV/ΔNS2/Δ1313/I1314L is generated by combining deletion of the NS2 gene and the 1313 codon in the L gene, with a stabilizing attenuation phenotype at codon 1314 by substitution of leucine with isoleucine in the RSV/A2 genome. The vaccine, administered intranasally, showed good attenuation and immunogenicity profile in clinical trials involving 6–24 month-old infants and children [172,173]. It has been shown that incorporating the four amino acid changes (I79M, K191R, T357K, N371Y) derived from the RSV strain “line 19” into the F protein of this candidate improved its stability and production scalability [174].

### 4.4. Recombinant Vector-Based Vaccines

Progress in recombinant virology has facilitated the design of vectored vaccines, including replicating and non-replicating viruses [175]. Adenoviruses and vaccinia virus vectors have been widely used in the development of recombinant RSV vaccines.

MVA-BN-RSV is an intramuscularly delivered candidate developed by Bavarian Nordic, based on the MVA (Modified Vaccinia Ankara)-BN backbone encoding the RSV F and G proteins and internal RSV N and M2 proteins [176]. This replication-defective vector effectively triggers viral protein expression without resulting in virion formation [177,178]. Phase 1 and phase 2 trials showed that MVA-BN-RSV elicits T-cells and antibody responses that persisted for 6 months and could be boosted at 12 months [179,180]. In a phase 3 study, vaccine efficacy was 42.9% against RSV-LRTD with ≥3 symptoms, 59.0% against LRTD with ≥2 symptoms, and 48.8% against ARD [181]. However, the primary end-point was not met for LRTD with ≥3 symptoms, leading to the discontinuation of the vaccine’s development.

Ad26.RSV.preF is another non-replicating vector based on adenovirus 26 encoding preF antigen, stabilized using unique amino acid substitutions focused on the stabilization of helix α4 in the disordered apex [182]. Intramuscularly administered Ad26.RSV.preF exhibited an acceptable safety profile in older adults, healthy adults, and toddlers and induced sustained humoral and cellular immune responses [183,184]. Its coadministration with the influenza vaccine for simultaneous seasonal vaccination revealed no interference in immune response [185]. A human challenge study revealed that immunization with Ad26.RSV.preF provides protection against RSV infection, indicating potential effectiveness against natural RSV infections [186]. The combination of the Ad26.RSV.preF vector with the stabilized RSVpreF protein as a vaccine (Ad26.RSV.preF–RSVpreF) yielded enhanced immunogenicity, with efficacy rates of 80%, 76.1%, and 78.7% against RSV-LRTD in adults ≥65 over the course of one, two, and three seasons, respectively [187,188]. Despite these promising results, Janssen decided to abandon the RSV adult vaccine program as part of its efforts to focus on other medical fields.

Developed by Blue Lake Biotechnology, BLB-201 is an intranasally delivered viral-vectored candidate based on a parainfluenza virus 5 (PIV5) encoding the full length of RSV-F antigen, designed to prevent infection in older adults and children under 2 years of age. The vaccine is undergoing phase 2 evaluation, and data from the phase 1 clinical trial exhibited satisfactory safety and immunogenicity profiles in RSV-seropositive adults with nasal RSV-specific IgA detected in 48% of participants [189].

### 4.5. mRNA Vaccines

mRNA vaccines consist of synthetic mRNA molecules coding for an antigen, which subsequently elicits an immune response following its translation in the host [190]. The COVID-19 pandemic urged the rapid development of the mRNA-LNP platform, which consists of lipid nanoparticles encapsulating mRNA molecules, for widespread human use. Research is ongoing to explore its applications against other pathogens [191]. The LNP formulation used in the SARS-CoV-2 vaccine SpikeVax^®^, which is known to induce and enhance antibody and T-cell responses, has been adapted for the development of an mRNA RSV vaccine that codes for the stabilized prefusion F protein of the virus [114]. mRNA-1345, namely mRESVIA^®^, is a single dose and intramuscularly delivered vaccine developed by Moderna. It contains 50 μg of pre-F mRNA and is designed to protect adults ≥60 years against RSV-LRTD [192]. Phase 1 evaluations showed that mRNA-1345 was well tolerated and immunogenic in young and older adults [193,194]. The Phase 2/3 ConquerRSV study evaluated the efficacy and safety of this vaccine in older adults ≥60 years (Table 1). They observed that the vaccine exhibited an efficacy of 83.7% in preventing RSV-LRTD, characterized by at least two symptoms, and 82.4% in cases with at least three symptoms. Additionally, it demonstrated a 68.4% efficacy against RSV-associated acute respiratory disease. The vaccine provided protection against both RSV subtypes (A and B) across different age groups and among individuals with pre-existing conditions [119]. These results led to the approval of the vaccine by the FDA in June 2024 in individuals 60 years of age and older [192].

Pediatric mRNA-based RSV vaccines have also been developed. A phase 1 study evaluated two dosing regimens (15 and 30 μg) of a monovalent RSV vaccine (mRNA-1345) and a bivalent RSV/HMPV vaccine (mRNA-1365) in healthy children aged from 5 months up to 24 months. In July 2024, the FDA notified a study pause in this phase 1 evaluation due to a safety concern: a potential increase for severe RSV-LRTI was observed with more cases identified in the vaccine groups compared with the placebo group, suggesting vaccine-associated enhanced respiratory disease (VAERD) [195]. Five cases of clinically significantly severe/very severe RSV-LRTI were reported in infants who received a dose of 15 μg of mRNA-1345 (2 cases) or 15 μg of mRNA-1365 (3 cases), compared to just one case in the placebo group. Among the six cases, five required hospitalization, including one infant who required mechanical ventilation. Moreover, the immune responses to the vaccine in infants who received mRNA-1345 (30 μg) and had previously been exposed to nirsevimab were decreased compared to those who had not, indicating a potential interaction. The FDA stated that the discrepancy in severe and very severe cases of RSV-LRTI among recipients aged 5 to under 8 months receiving 15 μg of mRNA-1345 or mRNA-1365 and placebo raises concerns for future development of non-living RSV pediatric vaccines. The trial was put on hold after those concerns were observed [195].

## 5. Combined Vaccines Including HMPV

Despite the fact that vaccine development for pneumoviruses has been primarily focused on RSV due to its greater burden compared to HMPV, significant efforts have been undertaken to develop vaccines for the latter since its discovery in 2001. The first HMPV vaccine tested in humans was a mucosal LAV in which the Phosphoprotein (P) gene was replaced by the one from its avian counterpart AMPV (rHMPV-Pa). In a phase 1 study, the rHMPV-Pa candidate successfully restricted viral replication in healthy adults and seropositive children but presented an over-attenuated profile in seronegative children as evidenced by illness occurrence in half of the vaccine recipients and HMPV-positive RT-PCR results, leading to its discontinuation [196]. At the time of this writing, no vaccine has been approved for HMPV. However, combined HMPV vaccine candidates that also target either RSV or HPIV3 or simultaneously both are currently under clinical trials (Table 2). 

Two bivalent candidates based on the mRNA technology, mRNA-1653 and mRNA-1365, have been developed by Moderna. mRNA-1365 targets RSV and HMPV, and its evaluation as a pediatric vaccine has been discussed in the RSV mRNA vaccines section. In addition to the enhanced respiratory diseases related to RSV, cases of HMPV hospitalization following infection have been reported in two vaccinees [195]. The second candidate, mRNA-1653, has been developed for the prevention of respiratory diseases associated with HMPV and HPIV3 infections. It represents the first combination mRNA vaccine simultaneously targeting these two viruses and encodes two distinct nucleoside-modified mRNA: the full-length fusion proteins of HMPV and HPIV3 co-formulated in an LNP [197]. Phase 1 evaluations in healthy adults and children aged 18–55 months demonstrated an acceptable safety profile, along with an increase in nAb titers for both viruses after one dose following intramuscular administration [197,198].

Sanofi Pasteur has developed F proteins-based mRNA vaccine candidates targeting RSV, HMPV and HPIV3. Trials assessing bivalent RSV/HMPV and trivalent RSV/HMPV/HPIV3 vaccines have been launched in 2024 [202,203,204,205].

IVX-A12 is a potential bivalent candidate targeting RSV and HMPV based on a virus-like particle (VLP) platform developed by Icosavax, recently acquired by AstraZeneca [199]. It includes two intramuscularly administered VLPs: RSV pre-F antigen (IVX-121) and HMPV pre-F antigen (IVX-241). Results from the phase 1 trial revealed an increase in nAbs titers for both viruses, with no safety concerns reported. Positive outcomes were observed from the phase 2 study in older adults with robust immune responses to all RSV and HMPV subgroups. IVX-A12 is phase 3-ready and has been granted Fast Track Designation from the FDA [199].

VXB-241 is a Vicebio Australia’s bivalent subunit candidate aiming to prevent RSV and HMPV infections. The vaccine was developed using a molecular clamp technology, consisting of a highly stable trimerization domain that, when incorporated into viral fusion proteins, effectively constrains them in their native prefusion conformation [206]. This technology has been used for the preclinical development of vaccines against diverse pathogens, including influenza A, SARS-CoV-2, and HPIV3 [207,208,209]. A phase 1 trial for VXB-241 started in 2024, with recruitment still in progress [200,209].

B/HPIV3/HMPV-PreF-A and B/HPIV3/HMPV-F-B365 are LAVs Bovine/Human Parainfluenza Virus Type 3 (B/HPIV3) vectored vaccines expressing the fusion proteins of HMPV. These two LAVs, developed by NIAID, are intended to protect against HMPV and HPIV3, and recruitment for the phase 1 study is still ongoing [201].

Among candidates in preclinical development, a mucosal bivalent LAV against HMPV and RSV developed by Vaxxel holds promise for preventing pneumovirus-induced diseases. The candidate was developed using an HMPV_ΔSH (Metavac^®^) backbone, which has demonstrated efficacy in protecting mice against HMPV infection following intranasal immunization [210]. Using this platform, the F gene of RSV/A2 was inserted alongside the endogenous HMPV-F gene, yielding the bivalent candidate Metavac^®^-RSV [211]. The latter efficiently expressed the exogenous RSV-F gene and replicated to high titers in vitro. Studies using the murine model demonstrated a good safety profile, strong immunogenicity, and protection against lethal challenges with RSV and HMPV [211]. Initiation of a phase 1 clinical trial should take place in 2026 [212].

## 6. Perspective

**Figure 2:**
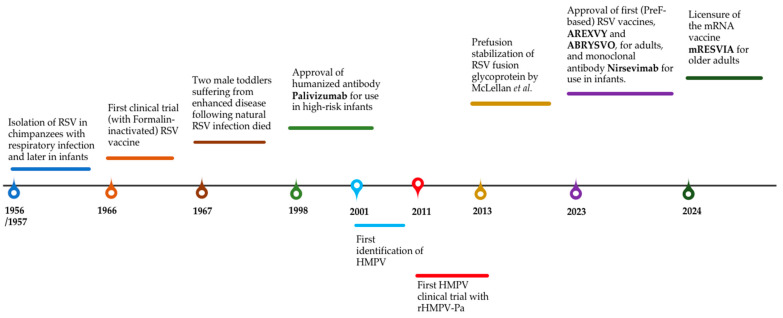
Pneumoviruses preventive modalities timeline.

Pneumoviruses are related to significant morbidity and mortality, justifying the need for preventive modalities, including vaccines (see timeline in Figure 2). The development of RSV vaccines has faced setbacks, especially with early inactivated vaccines, due to concern about vaccine-associated enhanced respiratory disease. The stabilization of the fusion protein in its prefusion form has marked a turning point in RSV vaccine research. It resulted in three vaccines now approved for use in older adults and pregnant women, demonstrating satisfying efficacy and safety results, with substantial economic and health benefits expected. However, it remains important to evaluate the long-term immunity provided by these vaccines over multiple seasons and the impact of revaccination, considering the seasonality of this virus. In addition, establishing standardized antibody thresholds would be essential to more accurately evaluate vaccine effectiveness and ensure consistent assessments.

The halt in Moderna’s pediatric mRNA vaccine study due to safety concerns related to VAERD highlights the complexity of vaccine development in this age group. Therefore, vaccine development should consider factors influencing immune response, such as age, pre-existing conditions, and prior exposure to the virus. Moreover, with a view to mitigating VAERD risk in seronegative children, preclinical studies should assess the four key immune properties recommended by WHO: (1) the ability to induce anti-RSV nAb, (2) the capacity to avoid generating non-nAbs and have a low binding IgG/nAb ratio, (3) the prevention of strong Th2-type CD4+ T cell response, and (4) ensuring the absence of alveolitis following a valid live RSV challenge. Coadministration studies with other vaccines have shown non-inferiority of immune responses, which is favorable for the successful rollout of vaccination programs.

Recently, there has been increasing interest in developing combination vaccines that target RSV, HMPV, and HPIV3 for a global reduction in the burden associated with these respiratory pathogens. These combination approaches are expected to offer broader protection and could eventually help mitigate a possible risk of HMPV and HPIV3 infections emerging as a result of RSV vaccination alone. Since the risk of VAERD is also associated with HMPV and HPIV3, it is crucial to establish specific parameters defining ERD related to these combinations, as well as identifying suitable vaccine immune properties that may help avoid this risk.

## 7. Conclusions

RSV and HMPV are recognized human pathogens responsible for life-threatening diseases with significant burdens in vulnerable groups, such as children and older adults. Efforts to implement prophylactic measures have led to the development of approved monoclonal antibodies and structure-based vaccines, which have proven their safety and efficacy in protecting against RSV-LRTI and severe LRTI. However, progress still needs to be made in the development of safe pediatric RSV vaccines, which could greatly reduce the impact of RSV-related diseases, particularly in low- and middle-income countries, where this virus remains a significant public health threat.

Moreover, greater emphasis should be placed on mucosal LAVs as they can elicit local immunity at mucosal sites while inducing robust and long-lasting systemic immune responses with reduced risk of priming ERD. Recent advances in combination vaccine design offer significant promise for the development of pan-pneumovirus vaccines that target both RSV and HMPV and, more broadly, pan-respiratory vaccines.

## Figures and Tables

**Table 1:** Phase 3 clinical trials of approved RSV vaccines.

Vaccine/Sponsor	Study	Participants	Immunization Scheme	Results	Refs.
***AREXVY* ^®^** **GSK**	Evaluation of efficacy against RSV-LRTD in older adults NCT04886596	Older adults (≥60 yrs.)	- RSVPreF3 Group: Administration of a Single dose of RSVPreF3 OA or placebo before RSV season	- Efficacy of 1 dose against RSV-LRTD: - 82.6% (96.95% CI, 57.9 to 94.1); - Efficacy of 1 dose against Severe RSV-LRTD: - 94.1% (95% CI, 62.4 to 99.9); - Efficacy of 1 dose against RSV-ARI: - 71.7% (95% CI, 56.2 to 82.3); - Median follow-up of 6.7 months - Efficacy consistent regardless of subtypes and condition.	[115]
	Evaluation of efficacy and Safety in older adults over 2 RSV seasons – NCT04886596	Older adults (≥60 yrs.)	- Extension of above study for year 2 - Second vaccine dose after 1 year (revaccination) or not	- Efficacy of 1 dose against RSV-LRTD over 2 seasons: - 67.2% (97.5% CI: 48.2–80.0%); - Efficacy of 1 dose against Severe RSV-LRTD over 2 seasons: 78.8% (95% CI: 52.6–92.0%); - Revaccination efficacy against RSV-LRTD: - 67.1% (97.5% CI: 48.1–80.0%); - Revaccination efficacy against Severe RSV-LRTD: - 78.8% (95% CI: 52.5–92.0%); - Median follow-up 17.8 months - Revaccination did not improve efficacy.	[116]
***ABRYSVO*** **^®^**	RSV Vaccine Efficacy Study in Older Adults Immunized against RSV Disease (RENOIR)– NCT05035212	Older adults (≥60 yrs.)	Administration of single dose (120 μg) of RSVpreF or placebo	- No evident safety concern reported; - Efficacy against RSV-LRTI with at least two signs or symptoms: 66.7% (96.66% CI, 28.8 to 85.8); - Efficacy against RSV-LRTI with at least three signs or symptoms: 85.7% (96.66% CI, 32.0 to 98.7); - Efficacy against RSV-ARI with at least two signs or symptoms: 62.1% (95% CI, 37.1 to 77.9).	[117]
	Maternal Immunization Study for Safety and Efficacy (MATISSE)–NCT04424316	Pregnant women at 24 through 36 weeks’ gestation	Administration of single dose (120 μg) of RSVpreF or placebo	- Efficacy against medically attended severe RSV-LRTI within 90 days after birth: 81.8% (99.5% CI, 40.6 to 96.3); - Efficacy against medically attended severe RSV-LRTI within 180 days after birth: 69.4% (97.58% CI, 44.3 to 84.1); - Efficacy against medically attended RSV-LRTI within 90 days after birth: 57.1% (99.5% CI, 14.7 to 79.8). - Effective against medically attended severe RSV-associated lower respiratory tract illness in infants, but criterion not met for medically attended RSV-associated lower respiratory tract illness (the statistical success criterion for this end point—lower boundary of the confidence interval > 20%—was not met).	[118]
***mRESVIA*^®^** **Moderna**	Evaluation of safety and efficacy of mRNA-1345 in preventing a first episode of RSV-LRTD in older adults (ConquerRSV) NCT05127434	Older adults (≥60 yrs.)	Administration of one dose of mRNA-1345 (50 μg) or placebo	- No evident safety concern reported in a median follow-up of 112 days; - Efficacy against RSV-LRTD with at least two signs or symptoms: 83.7% (95.88% CI, 66.0 to 92.2); - Efficacy against RSV-LRTD with at least three signs or symptoms: 82.4% (96.36% CI, 34.8 to 95.3); - Efficacy against RSV-ARD: 68.4% (95% CI, 50.9 to 79.7); - Protection consistent regardless of RSV subtypes, age, and conditions.	[119]

Abbreviations: LRTI = lower respiratory tract infection; LRTD = lower respiratory tract disease; CI = confidence interval; ARI = acute respiratory infection; ARD = acute respiratory disease.

**Table 2:** Combination vaccines, including HMPV, have entered clinical trials.

Vaccine/Sponsor	Formulation	Population Study	Phase 1	Phase 2	Results	Refs.
**- mRNA-1653/** **Moderna**	Two mRNAs encoding full-length of HMPV-F and PIV3-F co-formulated in LNP	Healthy adults aged 18–49 years	Dose selection phase, evaluation of safety and immunogenicity—NCT03392389	-	- Well tolerated - Maximum increase in nAbs titers reached with one dose - HMPV nAbs remained above baseline through month 13, whereas PIV3 nAbs titers declined at month 13 to baseline	[197]
		Children aged 18 to 55 months	Phase 1b, evaluation in seropositive children— NCT04144348	-	- Increase in nAbs titers GMF: HMPV/A = 2.9–6.1, HMPV/B = 6.2–13.2, PIV3 = 2.8–3.0 - Increase in bAbs titers GMF: HMPV preF = 5.3–6.1, HMPV postF = 4.6–6.5, PIV3 preF = 13.9–14.2, PIV postF = 11.0–12.1 - Binding antibody responses were generally preF biased, and no benefit of a second dose	[198]
**- mRNA-1365/** **Moderna**	mRNAs encoding RSV-F stabilized in the preF conformation and HMPV-F, encapsulated in LNP	Children aged 5 months to <24 Months	Evaluation of safety, tolerability, and immunogenicity—NCT05743881	-	- Imbalance in the number of clinically significant severe/very severe LRTI reported in the vaccine group: - Three patients aged 8 months developed severe RSV-LRTI with one very severe case - Two HMPV hospitalizations reported	[195]
**- IVX-A12/** **Icosavax, Inc.**	Bivalent combination Of RSVpreF-VLP (IVX-121) and HMPVpreF-VLP (IVX-241)	Older adults	Evaluation of safety and immunogenicity of three dosage levels (low, medium, high)—NCT05664334	Phase 2a-Evaluation of immunogenicity and safetyNCT05903183	- Well-tolerated - Phase II data demonstrate that IVX-A12 elicits robust immune responses against both RSV and HMPV one month after vaccination and reconfirm previous immunogenicity data seen in the phase I trial.	[199]
				Phase 2-Characterization of immunogenicity and safetyNCT06481579		
**- VXB-241/** **Vicebio Australia**	Molecular Clamp Stabilized Prefusion F Glycoprotein Subunit Bivalent RSV and HMPV	Older adults With Run-in in Young Adults	Evaluation of safety, reactogenicity, and immunogenicity of four dose levels—NCT06556147	-	Recruiting	[200]
**- B/HPIV3/HMPV-PreF-A** **- B/HPIV3/HMPV-F-B365** **NIAID**	Two recombinant LAVs B/HPIV3 vectors expressing fusion proteins of HMPV	Children 24 to <60 Months of Age	Study of the infectivity, safety, and immunogenicity in HPIV3-Seropositive children—NCT06546423	-	Recruiting	[201]
**- RSV/HMPV** **Sanofi Pasteur**	Bivalent RSV/HMPV mRNA-LNP	Older adults (≥60 yrs.)	Phase 1/2a, evaluation of safety and immunogenicity— NCT06134648		Ongoing	[202]
		Older adults (60–75 yrs.)	Evaluation of safety and immunogenicity—NCT06583031	-	Recruiting	[203]
		Participants aged 18 to 49 years and 60 years and older	Evaluation of safety and immunogenicity—NCT06237296	-	Ongoing	[204]
**- RSV/HMPV/PIV3** **Sanofi Pasteur**	Combined RSV, HMPV, PIV3 mRNA vaccine	Older adults (≥60 yrs.)	Evaluation of safety and immunogenicity—NCT06604767	-	Recruiting	[205]

Abbreviations: nAbs = neutralizing antibodies; bAbs = binding antibodies; GMF = geometric mean fold; preF = prefusion; postF = postfusion; LRTI = lower respiratory tract infection; LNP = lipid nanoparticle.

## Data Availability

Not applicable.
